# Internal and Predictive Validity of the French Health of the Nation Outcome Scales: Need for Future Directions

**DOI:** 10.1371/journal.pone.0160360

**Published:** 2016-08-02

**Authors:** Philippe Golay, Louis Basterrechea, Philippe Conus, Charles Bonsack

**Affiliations:** Department of Psychiatry, Lausanne University Hospital (CHUV), Lausanne, Switzerland; University of Manchester, UNITED KINGDOM

## Abstract

The Health of the Nation Outcome Scales (HoNOS) is a widely used measure of health and social functioning of people with mental illness. The goals of this study were to verify the internal validity of the one factor and several four-factor scoring structures and to evaluate the predictive validity of HoNOS items with regards to duration of hospitalization, probability of readmission in the following year and time before readmission. 6175 hospital stays at the department of psychiatry of Lausanne University Hospital were screened and the first HoNOS of each patient was taken into account (N = 2722). Data were analyzed through Confirmatory Factor Analysis (CFA) and the predictive validity of HoNOS items was evaluated with two approaches: item level regressions and latent class analysis (LCA). CFA indicated that the suggested factor structures were not supported by the data. Predictive validity of the 12 items was weak but LCA revealed five distinct and meaningful profiles that were related to length of stay or readmission. HoNOS may be more adapted to the evaluation of patients case-mix rather than to the individual level and concepts such as predictive validity may be more appropriate than internal validity to guide its use.

## Introduction

The Health of the Nation Outcome Scale (HoNOS) was first developed in the context of the UK’s Health of the Nation strategy in order to quantify improvement of health and social functioning of mentally ill people [1,2]. The HoNOS is composed of 12 likert scales that measure health and social functioning and was developed over multiple phases between 1992 and 1995 [1]. Adaptation for children and adolescent and for older people were later developed [3,4]. The HoNOS and its variant are being extensively used as routine clinical outcome measures in the UK [2] and in other countries like Australia [5], Switzerland [6,7] or Canada [8].

The internal validity of the HoNOS scales has often been questioned. It has been argued that the HoNOS scales did not measure a single underlying dimension and that the initial four factor solution was not supported [7,9–12]. The validation of the French version was nevertheless conducted on a rather modest sample [7]. The unidimensionality of HoNOS was also found to be irrealistic in an Italian study: only 6 items out of 12 could be retained to form a unique clinically acceptable outcome scale [13]. Other variants based on four [11] or five factors also failed to achieve acceptable fit to the data [9,11]. It is worth noting that other confirmatory factor analysis focusing on the older adults version (HoNOS65+) also failed to support any suggested structures [14]. Two factor solutions that did not include all 12 items were also suggested [15,16]. A modified 4 factor structure was also proposed by Newnham and colleagues [17] and was both supported by the data and successfully replicated on a second sample. The two studies were however conducted on the same site and this model has still to achieve wider acceptance. A recent large sample study on the original version of the HoNOS demonstrated that no factor structure could be considered as adequate with regards to model fit even if a modified four-factor model could still be favoured on a “best fit” rather than “good fit” basis [18]. This study was followed by a new large confirmation study acknowledging the ordinal nature of the HoNOS items. It was concluded that while superior to other structures, this solution was less than optimal for some clinical populations and that additional items should be identified and added to allow for a unique, more generalizable and improved version of the HoNOS [19]. Because many studies have failed to demonstrate or replicate an adequate factorial structure, the question whether HoNOS items should even be grouped into one or several sub-dimensions remains open to scrutiny. Indeed, the lack of internal validity of HoNOS could be explained so far by the fact that the 12 HoNOS items simply cannot be reasonably explained by fewer underlying dimensions.

Numerous studies have focused on concurrent validity with other established instruments with generally favorable results [2]. The conclusiveness of studies exploring the predictive validity of the HoNOS with resource use and treatment outcome is nevertheless more contrasted: on the one hand HoNOS was identified as the most distinctive predictor of outcome in studies [20] and correlated, among others, with depression, quality of life and number of contact with service [21]. On the other hand, while significant differences between admission and discharge for total and sub-scales scores were found, there were either no significant associations or only very weak associations of doubtful practical clinical significance between HoNOS scores and length of stay [22]. The same authors concluded that while the HoNOS provided a readily administered and understood measure for clinicians, “*it was unlikely to be of utility in predicting length of stay or in offering a 'gate-keeping' service in decision-making in regard to the allocation of resources for individual patients*” (p.199). In Denmark the total HoNOS score was nevertheless found valid to discriminate between frequently and non-frequently hospitalized patients [23]. In another study, HoNOS proved worthwhile to characterize case-mix but only modestly contributed to prediction of length of stay [24]. Finally considering that the validation study on the French version (HoNOS-F) was conducted on a relatively small sample (N = 194) and did not include concurrent validity evidence [7], its validity evidence in relation with resource use could still be considered as weak or uncertain.

Considering that HoNOS is extensively used to define case-mix and measure differences in functioning between admission and discharge, we designed the current study in order to verify the internal validity of a one and a four factor scoring structure of the HoNOS-F using recent psychometric methods in a large patient sample. We also included the Newnham et al. [17] and Speak et al. [18,19] modified four factor structures because there were replicated in large samples but needed replication in another sites, settings or languages. The first objective of this study was to establish whether the HoNOS-F items were suitable for construction of synthetic indicators of severity or whether an item-level approach was more appropriate. Acknowledging that predictive validity is one of the most important forms of validity, we also wanted to gather additional evidence concerning the predictive validity of the HoNOS-F with indicators of service use. Therefore the second objective of this study was to evaluate the association between HoNOS-F items and duration of hospitalization, probability of psychiatric readmission and time to readmission. For this purpose, analyses were performed at both the individual level (regression models) and at the group level (“case-mix” approach with specific patient profiles using latent class regression models).

## Material and Methods

### Sample and setting

HoNOS-F subscales are routinely assessed at any hospital admission (voluntary or involuntary) and at discharge at Lausanne University Hospital’s psychiatry department (DP-CHUV) since June 2010. Research involved audit of anonymised routine institutional records. The only identifiable data that were extracted were the patients’ identification numbers. They were irreversibly transformed by the addition of a randomly selected unknown constant. This code allowed identification of duplicate entries for a given patient but rendered identification impossible for the authors. While unlikely, it was therefore not possible to determine whether the authors were directly involved in the treatment of the individuals included in this retrospective study. Local ethics committee approval and explicit consent were not required because analyses were conducted on existing records containing anonymous data. A total of 6175 hospital stays in adult psychiatry between June 2010 and September 2013 were screened for the purpose of this study. Analyses were restricted to the first HoNOS assessment conducted for each patient over the entire study period (data from repeated admissions were discarded), thus reducing the number of entries to 3088. To ensure data quality, 366 entries were excluded: 271 entries contained no HoNOS data, 89 entries included missing items that were not coded as “unknown” revealing deviation from the correct scoring procedure, 6 entries contained all twelve items quoted as “unknown” providing no information.

### Measures

The French version of Health of the Nations Outcome Scales (HoNOS-F) is similar to other language versions of the HoNOS scales and is composed of 12 scales / items measuring frequent problems in patients with mental health problems [7]. During the developpment of the French version, each statement was tested for readability by seven experts. The scale was then administered to three samples of people suffering from severe mental disorders to estimate its internal consistency, test-retest and interrater reliability. Each item or scale is quoted on a Likert-type scale from 0 (*no problems during the reporting period*) to 4 = (*severe to very severe problem*). When information is not sufficient, raters indicate “unknown” for the corresponding items. The twelve items are grouped in four sub domains [1] such as *Behavior*, *Impairment*, *Symptoms* and *Social* (Table 1). The Newnham modified four factor structure [17] included an *anti-social* factor (item 1, 2, 3 & 9), an *impairment* factor (items 4, 5, 6 & 10), a *symptom* factor (items 7 & 8) a *socio-economic factor* (items 11 & 12) and correlated error terms between three pairs of items (items 1 & 7, items 5 & 6, items 9 & 10). The Speak modified for factor structure [18,19] included a *personal well-being* factor (item 4, 5, 10 & 12), a *social well-being* factor (items 3, 9, 11, 12) an *emotional well-being* factor (items 2, 7 & 8), a *severe disturbance factor* (items 1 & 6) and three correlated residuals (item 2 & item 8, item 4 & item 7, item 6 & item 7).

**Table 1:** Loadings for the one-factor Confirmatory Factor Analysis (N = 2722).

		Loadings	Residual variance (item specific) %
	Subscale	Factor I	
Items			
1 Overactive, aggressive, disruptive or agitated behaviour	a	0.262*	93.2
2 Non-accidental self-injury	a	-0.101*	99.0
3 Problem drinking or drug-taking	a	0.022	100.0
4 Cognitive problems	b	0.512*	73.8
5 Physical illness or disability problems	b	0.247*	93.9
6 Problems associated with hallucinations and delusions	c	0.353*	87.5
7 Problems with depressed mood	c	-0.001	100.0
8 Other mental and behavioural problems	c	0.139*	98.1
9 Problems with relationships	d	0.627*	60.7
10 Problems with activities of daily living	d	0.817*	33.3
11 Problems with living conditions	d	0.501*	74.9
12 Problems with occupation and activities	d	0.685*	53.0

Note.

*p<.05.

a = Behavior subscale; b = Impairment subscale; c = Symptoms subscale; d = Social subscale.

### Statistical Analysis

Items that were scored as “unknown” were treated as missing data in all analysis. To test the adequacy between *a priori* item grouping and empirical data, confirmatory factor analyses (CFA) were conducted. In order to verify the relevance of a total score of severity, a one factor model was estimated. To evaluate the relevance of a model based on four sub-scores (*Behavior*, *Impairment*, *Symptoms and Social*) [1] a four factors model was also tested. Data were treated as categorical ordinal and models were estimated using a robust weighted least squares estimator with adjustments for the mean and variance (WLSMV). Several indicators of model fit were used such as the root mean square error of approximation (RMSEA), the Comparison fit index (CFI) and the Tucker–Lewis fit index (TLI). RMSEA less than .06 and CFI and TLI larger than .95 are interpreted as good fit while values of RMSEA ≤.08 and CFI/TLI ≥ .90 are often considered as indicating acceptable fit [25]. To the best of our knowledge interpretation of global fit indexes in models with ordered categorical indicators is not as well established as it is with continuous indicators [25]. While simulation studies suggest that these cut off values works reasonably well with categorical outcomes [26,27], the exact cut-off scores may not perfectly apply in the context of our study.

To evaluate the predictive validity of HoNOS items, a series of stepwise linear regression for continuous outcomes (duration of hospitalization & time to readmission) and logistic regression for binary outcomes (readmission within the next year) were performed. In each case, the twelve HoNOS items were entered as independent variables.

To assess the existence of specific patients HoNOS’ profiles, a latent class analysis (LCA) was conducted. HoNOS items were first dichotomized into "*no serious problem*" (quotations 0, 1 & 2) and "*severe problem*" (quotation 3 & 4) to facilitate model estimation. The best solution was determined using the BIC (Bayesian Information Criterion) coefficient [28]. The BIC balance model fit with its complexity (number of parameters). For the sake of parsimony, it was further verified whether a solution with one less class could present a similar degree of adjustment. In this regard, a Lo-Mendell-Rubin Adjusted Likelihood Ratio Test and a Parametric Bootstrapped Likelihood Ratio Test were performed.

Finally, the relationship between such profiles and the three observed distal outcomes (length of stay, likelihood of rehospitalization within the next year & the period before readmission) was estimated using a 3-step latent class regression model with the Lanza method for continuous or categorical distal variables [29,30]. All statistical tests were two-tailed and significance was determined at the .05 level. All statistical analyses were performed with the Mplus statistical package version 7.3 and IBM SPSS version 22.

## Results

The final sample included 2722 patients with complete HoNOS data at admission. The sample mean age was 38.8 years (SD = 12.2) and 53.3% male. Final primary diagnoses based on ICD-10 criteria were 24 Dementia (0.9%), 287 Alcohol use (10.5%), 435 Drug use (16.0%), 649 Schizophrenia (23.8%), 176 Mania (6.5%), 587 Depression (21.6%), 230 Anxiety and stress related disorder (8.4%), 49 Behavioral syndromes associated with physiological disturbances and physical factors (1.8%), 198 personality disorder (7.3%) and 87 without diagnostic information at discharge (3.2%).

### Internal validity of the HoNOS

Results of the one factor CFA model are presented in Table 1. Several items showed very moderate or not significant loadings indicating that a number of domains assessed by HoNOS did not contribute to a general dimension. Examination of percentages of residual variances also revealed that most items were only very partially explained by a single general factor of severity.

As shown in Table 2, this model showed an unacceptable degree of fit to the data. Taken altogether these elements led to the conclusion of the inadequacy of a global composite score summarizing the 12 HoNOS items.

**Table 2:** Comparison of model fit for the Confirmatory Factor Analysis (N = 2722).

Models	χ2	df	p-value	RMSEA	90% C.I RMSEA	CFI	TLI
1 factor model	1847.281	54	<.001	0.110	0.106–0.115	0.701	0.634
4 factor model	979.674	48	<.001	0.084	0.080–0.089	0.845	0.786
4 factor Newnham et al. model	1357.773	45	<.001	0.104	0.099–0.108	0.781	0.679
4 factor Speak et al. model	6414.783	44	<.001	0.071	0.066–0.076	0.900	0.850

Note. df = degrees of freedom; RMSEA = Root Mean Square Error of Approximation; C.I = Confidence Interval; CFI = Comparative Fit Index; TLI = Tucker-Lewis Index.

The estimation of the 4-factor model revealed to be problematic: while the model estimation terminated normally, the latent variable covariance matrix was found to be not positive definite. Examination of the inter-item polychoric correlation matrix revealed that the first three items did not correlate with each other making the definition of a “*behavior*” factor on the basis on their common variance illusive. Thus, reliable estimates of the loadings of this first factor were not available (Table 3). Therefore it was also not possible to obtain reliable estimates of the correlation between this first factor and the three other factors. Despite this, examination of the loadings on other factors revealed some cohesion between items for the *Impairment* and the *Social* factors. Concerning the *Symptoms* factor however, the expected factor loadings pattern was not demonstrated because a non-significant loading (item 8) and two loadings of opposite signs (6 and 7). Examination of fit indices (Table 2) also indicated an inadequate degree of fit to the data of this four sub score variant. All these elements supported unequivocally the inadequacy of the four-factor solution. The Newnham modified four factor structure also proved to be inadequate because of a non positive definite covariance matrix and poorer model fit than the original four factor structure. Finally the Speak modified four factor structure was estimated. While this solution unequivocally provided the best model fil overall (Table 2) adjustement could not considered as “good”, three factor loadings were not significant (item 12 on the *personal well-being* factor, item 3 on the *social well-being* factor & item 8 on *the emotional well-being* factor) and the latent variable covariance matrix was found to be not positive definite.

**Table 3:** Loadings for the four-factor Confirmatory Factor Analysis (N = 2722).

	Loadings				Residual variance (item specific) %
	Factor I Behavior	Factor II Impairment	Factor III Symptoms	Factor IV Social	
Items					
Item 1	N/A	0	0	0	100.0
Item 2	N/A	0	0	0	100.0
Item 3	N/A	0	0	0	100.0
Item 4	0	0.971*	0	0	5.7
Item 5	0	0.339*	0	0	88.5
Item 6	0	0	0.660*	0	56.4
Item 7	0	0	-0.470*	0	77.9
Item 8	0	0	-0.047	0	99.8
Item 9	0	0	0	0.625*	60.9
Item 10	0	0	0	0.831*	30.9
Item 11	0	0	0	0.514*	73.6
Item 12	0	0	0	0.695*	51.7
Factors	Factor correlations				
	I	II	III	IV	
I	1.00				
II	N/A	1.00			
III	N/A	0.279*	1.00		
IV	N/A	0.489*	0.157*	1.00	

Note.

* p<.05;

N/A = not estimable.

### Predictive validity of the HoNOS

The results of the duration of hospitalization linear regression model revealed that only four items were related to this outcome: Item 6 (*Problems associated with hallucinations and delusions*; β = .115, p < .001), item 10 (*Problems with activities of daily living*; β = .096, p < .001), item 4 (*Cognitive problems*; β = .072, p = .002) & item 12 (*Problems with occupation and activities*; β = .053, p = .035). Examination of the percentage of explained variance (R^2^) revealed that only 5.0% of the total variance could be explained by the HoNOS items.

The results of the rehospitalization within 365 days logistic regression model indicated that only two items were related to this outcome: Item 6 (*Problems associated with hallucinations and delusions*; B = 0.114, Odd ratio = 1.121, p = .002) and item 3 *Problem drinking or drug-taking*; B = 0.092, Odd ratio = 1.097, p = .006). Effect size indicated that the predictive validity of HoNOS items was very modest (Nagelkerke’s R^2^ = .012).

The results of the time before re-hospitalization linear regression model revealed that only two items could predict this outcome: Item 11 (*Problems with living conditions*; β = -.172, p < .001) and item 7 (*Problems with depressed mood*; β = .084, p = .046). Examination of the percentage of explained variance (R^2^) revealed that only 3.5% of the total variance could be explained by the HoNOS items.

### HoNOS-based profile analysis

Characteristics from one to eight classes LCA are presented in Table 4. The five-class solution was preferred on the basis of its lowest BIC and clinical interpretability. For the sake of parsimony, it was verified whether a solution with one less class could present a similar degree of adjustment. In this regard, a Lo-Mendell-Rubin Adjusted Likelihood Ratio Test and a Parametric Bootstrapped Likelihood Ratio Test indicated that a five class solution was preferable to a four class solution (p = .037 respectively p < .001).

**Table 4:** Characteristics of the 1–8 class latent class analysis solutions.

Number of classes	Size of the smallest class	Entropy	BIC
1	2722	-	35161.559
2	874	0.681	33795.184
3	760	0.659	33473.256
4	339	0.737	33415.776
5	172	0.737	33406.039
6	190	0.736	33417.082
7	140	0.731	33457.971
8	71	0.717	33505.831

Note. BIC = Bayesian Information Criterion.

**Fig 1:**
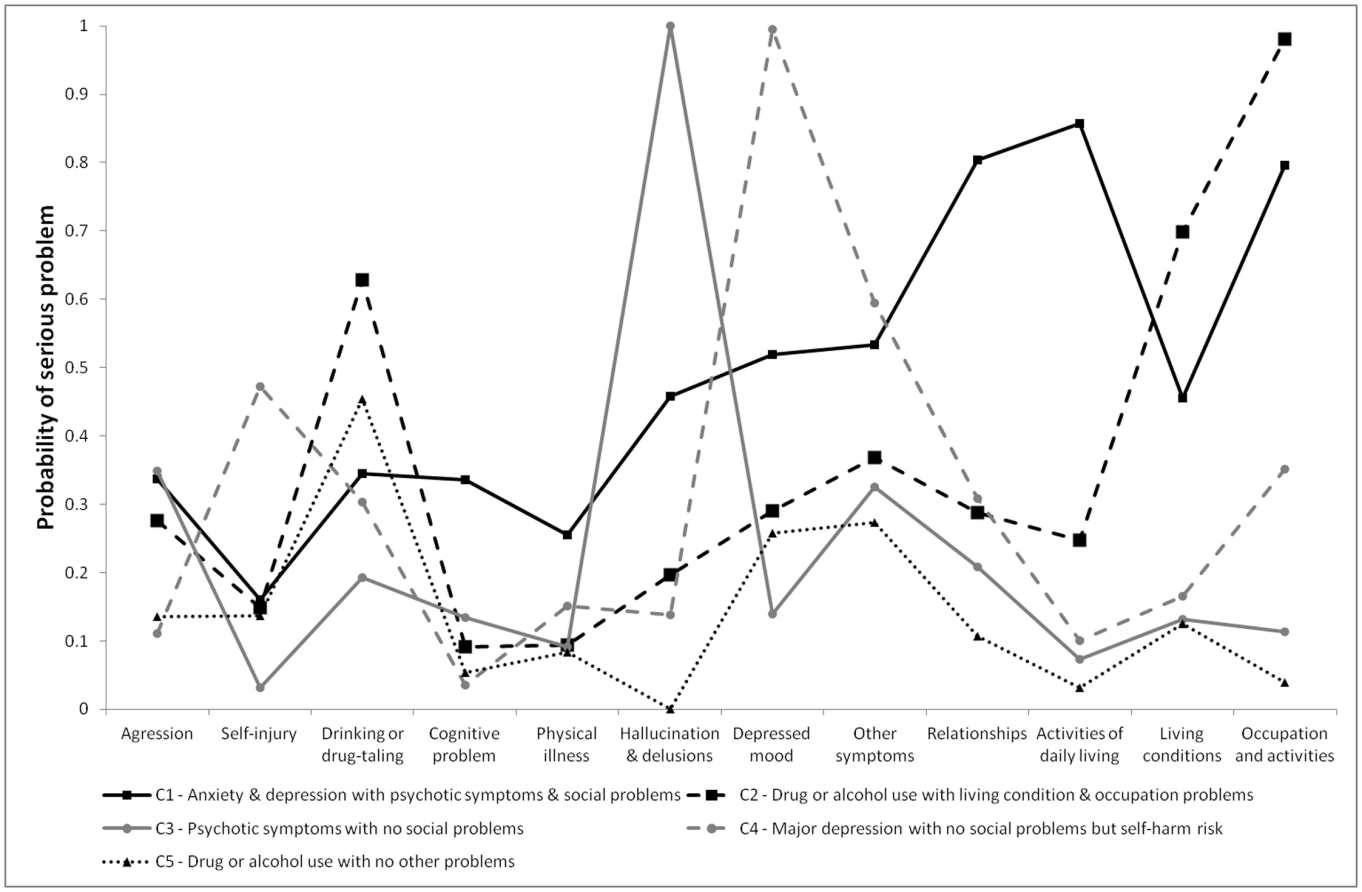
Probability of having serious problem in HoNOS items within five latent classes.

The characteristics of the five-class solution are represented in Fig 1. The first class (20.3% of the sample) consisted of patients presenting anxiety and depression problems with psychotic symptoms and significant social problems. The second class (8.0% of the sample) consisted of patients with problematic drug or alcohol use and important living condition and occupation problems. The third class (12.7% of the sample) included patients with psychotic symptoms but no social problems. The fourth class (23.9% of the sample) consisted of patients with a major depression with no social problems but self-harm risk. The fifth class (35.2% of the sample) included patients with drug and alcohol use with no or mild other problems.

Membership in one of the five classes was associated with different durations of hospitalization (χ^2^ = 165.047, df = 4, p < .001). Longest to shortest duration of hospitalization in days were distributed as such: the first class (anxiety and depression problems with psychotic symptoms and significant social problems; Mean = 37.1, SE = 2.3), second class (problematic drug or alcohol use and important living condition and occupation problems; Mean = 31.0, SE = 2.5), third class (psychotic symptoms but no social problems; Mean = 29.7, SE = 1.9), fourth class (major depression with no social problems but self-harm risk; Mean = 21.5, SE = 0.7) and fifth class (drug and alcohol use with no or mild other problems; Mean = 16.4, SE = 0.4).

Membership in one of the five classes was also associated with different probabilities of rehospitalisation within the next year (χ^2^ = 10.114, dl = 4, p = .039). Highest to lowest probabilities were distributed in the following way: third class (35.2%, SE = 3.1), first class (30.7%, SE = 2.9), second class (29.7%, SE = 5.5), fifth class (26.5%, SE = 1.7) and fourth class (24.4%, SE = 2.3).

Membership in one of the five classes was finally associated with different duration before rehospitalization (χ^2^ = 39.663, dl = 4, p < .001). Shortest to longest durations before rehospitalization in days were organized as such: second class (Mean = 106.6, SE = 17.4), first class (Mean = 168.1, SE = 13.2), fourth class (175.5, SE = 16.3), third class (Mean = 194.0, SE = 23.0) and fifth class (Mean = 246.6, SE = 14.7).

## Discussion

To the best of our knowledge, this is the first large scale study simultaneously examining internal and predictive validity evidence of the HoNOS-F scales with recent psychometric methods (CFA for categorical indicators and LCA).

Results of the CFA revealed that the one and four-factor alternatives did not fit well to the data. HoNOS items were only anecdotally explained by a single general factor of severity. The *behavior* factor was not supported by the data. While adequate loadings were found for the *impairment* and the *social* factors, results suggested that items related to the *symptoms* factor were not determined by a single underlying dimension. Additionnaly the goodness of fit of the Newnham modified four factor structure was even less adequate than the original structure. Similarly as observed in the initial study, the Speak four factor variant could be considered as the best fit rather than “good fit” [18]. Additionaly three items did not correspond to the *a priori* structure. In conclusion and in line with previous studies [7,10,12], it appeared that HoNOS total and sub-scores had poor internal validity. It could be argued that the reason so many studies failed to propose adequate factor structure may not reside in the fact the perfect structure has to be found yet but rather in the nature of the variables in use. A very strong postulate of the factorial approach is that every item within a factor is causally determined by a single underlying dimension (i.e reflective variable). As stated by Chin [31]”*an underlying assumption for Structural Equation Modeling (SEM) analysis is that the items or indicators used to measure an latent variable are reflective in nature*. *Such items are viewed as affected by the same underlying concept (i*.*e*., *the latent variable)*. *Yet a common and serious mistake often committed by researchers is to inadvertently apply formative indicators (also known as cause measures) in an SEM analysis*. *Formative indicators*, *first introduced by Blalock (1964)*, *are measures that form or cause the creation or change in an latent variable*. *An example is socio-economic status (SES)*, *where indicators such as education*, *income*, *and occupational prestige are items that cause or form the latent SES variable*. *(…) formative indicators need not be correlated nor have high internal consistency such as Cronbach's alpha*”. We should therefore question if the HoNOS scales are compatible with such assumptions. For instance one can wonder wheter a latent *Behavior* dimension will causally simultaneously influence *Overactive*, *aggressive*, *disruptive or agitated behavior*, *non-accidental self-injury and Problem drinking or drug-taking*. On the basis of several inconclusive studies and our findings, HoNOS items are likely not suitable to the construction of synthetic indicators of severity by means of reflexive latent variables; therefore the quest for the perfect factor structure may well be illusory. In conscequence each item could rather be used as a separate dimension. This recommendation is perfectly in line with other studies conducted on the German version of the HoNOS scales [9] or the Dutch version of the HoNOS 65+ [14] who supported item-level rather than global score use of the HoNOS scales. Concerns have been raised about considering HoNOS as 12 non-interdependent scales because the reliability of single-item scales can be legitimely questioned [11] and because the 12 HoNOS items cannot be considered as uncorrelated [16]. However, since validity implies reliability [32,33] the choice of the most relevant HoNOS items could be based on pragmatic grounds including their predictive value for relevant outcomes. We therefore want to underline the utmost importance of external validity in the choice of the most adequate HoNOS scores, some of them should be correlated or not with each other. Adequate HoNOS scores should not be selected to internal validity evidence alone.

The results of the three regression models for predicting duration of hospitalization, probability of psychiatric readmission in the following year and time to readmission highlighted some important HoNOS items Some element replicated previous findings: for instance, the important role played by Substance use regarding the risk of readmission was already identified in so called “revolving door” patients [34]. However, it should be noted that the various outcomes we explored (duration of hospitalization, risk of readmission and delay until readmission) were not predicted by the same dimensions. While our data provide concurrent validity evidence that was currently laking for the HoNOS-F, important concerns remains about the scale: the percentage of explained variance for each of the three outcomes was very small, calling into question the predictive validity of HoNOS-F scores in this particular context. Our results are in line with other studies [22,24] based at the individual level that concluded at the limited utility of HoNOS scores concerning prediction of service use. Despite these convergent findings, the HoNOS scales were not specifically designed to predict duration hospitalisation and readmission. Therefore one can wonder whether too much is expected from the HoNOS scales? On the one hand a recent study including 738 inpatients found length of stay not to be predictable to any practical degree by various patients characteristics [35]. One the other hand the initial goal of HoNOS was to quantify improvement of health and social functioning of mentally ill people. Such an undertaking may prove impossible if health care providers cannot rely on HoNOS data for decision making and predicting important dimensions. In order to explore new alternatives, one can also make a case for PARADISE 24 [36] which has been developed on more solid psychometric and conceptual grounds in order to measure the impact of brain disorders on people’s lives. The items of this scale have been based on the psychosocial difficulties that are experienced in common across brain disorders but could very likely be tested and used in psychiatric settings.

Considering the poor predictive validity of items, we decided to explore the existence of specific patient profiles that could be differentially related to duration of hospitalization, probability of psychiatric readmission in the following year and time to readmission. Results of the LCA suggested that patients could be divided into five characteristic profiles. In line with the Case-mix approach, patients could be clustered into meaningful and relatively homogeneous classes on the basis on their twelve HoNOS-F item scores. Interestingly, this classification suggested that patients with substance use could be divided into two distinct subgroups depending on the presence or not of concurrent important living condition and occupation problems. Similarly, patients with psychotic symptoms could be sub-divided into two distinct categories depending on the presence or not of concomitant important social problems. The latter category was also characterized by more frequent anxiety and depression problems. This suggests that co-morbidity with mood disorders could be associated with very increased likelihood of social problems. Finally, a last category also including patients with depressive symptoms was highlighted. While the occurrence of social problems was very low, they presented the highest risk of self-harm of the five categories.

The question whether similar or different profiles could be found in other instutitional settings remains open. It appears relatively likely that classifications based on very different case-mixes could yield different profiles. What appeared most important to us is that categories established on the basis of all items are associated with distinct outcomes. Such categories gather less precise information on several distinct dimensions and could therefore give a concise yet more comprehensive overview of a group of individuals. For instance, in our setting, membership in one or the other of these profiles was differentially related to duration of hospitalization, probability of psychiatric readmission and time to readmission. This highlighted the fact that it was possible to perform HoNOS scores based predictions through this process of group categorization rather than at the individual level. The two classes that involved longer hospitalization durations had higher prevalence of daily living problems (combination of *activities of daily living*, *living conditions & occupation*-*activities*). The severity profile of the class with shorter hospitalization duration was especially mild with only drug or alcohol use issues. Interestingly, examination of other items probabilities did not reveal housing problems that may have been a drive towards hospitalization. This phenomenon was previously identified by Bech and colleagues [23] who reported that patients with higher scores on drug and alcohol use tended to have shorter stay. It has been hypothesized that patients with dual diagnosis (i.e schizophrenia and substance use disorder) were more likely to be hospitalized for the misuse rather than the underlying mental condition [23,34].

Patients who were most likely to experience psychiatric readmission had psychotic symptoms but no social problems. The mean duration of hospitalization of this group of patients was not particularly long in comparison with other profiles. It could be hypothesized that the severity of their situation may often have been overshadowed by their apparent unproblematic social situation, resulting to a too short hospitalization likely followed by a readmission at some point during the following year.

Interestingly, profiles that were more likely to be rehospitalized were not always associated with shorter time before readmission. While patients presenting anxiety and depression problems with psychotic symptoms and significant social problems were likely to be rehospitalized within a relatively short interval, patients with problematic drug or alcohol use and important living condition and occupation problems spent the shortest interval between hospital stays if readmitted but were not more likely to be readmitted within the year.

In many cases HoNOS data are collected as part of routine clinical practice. This work is often seen as superfluous on the clinical level but could be used to prompt clinical practice and aid in institutional decision making [37]. Indeed and in line with the results of the present study, HoNOS items could be used in a first step to identify specific subgroups within a population. In a second step, specific profile membership and its association with important variables could be used to inform clinical and instutitional decisions.

Some limitations of our study should be mentioned. First, the range of outcomes was limited to three important dimensions that did not include costs or detailed service information. Second, while our study was based on a relatively large highly representative sample of our catchment area, it may suffer from bias inherent to unique site methodology. Third, HoNOS assessment was conducted by numerous clinicians and inter-rater reliability was not assessed, considering the data was not collected in a research setting. However, it is interesting to see that despite this limitation, the data collected allowed the identification of relevant subgroups regarding the outcomes we had chosen.

### Conclusion

Taken together, the results of the present study suggest that the individual HoNOS sub-scales are not suitable for the construction of synthetic indicators of severity. While it is more appropriate to use item scores based on forecasting capabilities evidence, predictive validity of the twelve HoNOS items was very weak. However, the case-mix approach seems more promising, allowing patient categories to be established on the basis of all items, which were differentially associated with duration of hospitalization, probability of psychiatric readmission and time to readmission. Traditional clinical scales are designed to gather individual information on narrow dimensions and rely on concepts like internal and external validity. They also assume that every item within a factor is causally determined by a single underlying dimension. HoNOS scales probably do not rely on such assumptions and may be more adapted to the assessment of case-mix rather than the individual level.
